# Supplementary material for: Population structure reverses selection of variants with proportionally scaled birth and death rates
Source: Nat Commun. 2025 Dec 27;17:244. doi: 10.1038/s41467-025-66951-x (PMC12783849; doi:10.1038/s41467-025-66951-x)
Supplement: Supplementary file 1 — Supplementary Infomation [file 41467_2025_66951_MOESM1_ESM.pdf]

Population structure reverses selection of variants with proportionally scaled birth and death rates

Supplemental information

Supplementary Note 1. Probability of mutant fixation in a fragmented population: a coarse-grained approximation

1.1. Preliminaries

1.1. Set-up and notations

Suppose the dynamics of two species (the wild-type and the mutant) are described by a death and birth process (e.g. a stochastic variant of the Verhulst process [1, 2], such as studied in [3]).

Let us denote by r_w and d_w the the intrinsic, per capita wild-type division and death rates of wild-type individuals, and by r_m and d_m the intrinsic, per capita division and death rates of mutants. We assume that

$$r_w > d_w, \quad r_m > d_m. \tag{1}$$

Here we will study the probability of mutant fixation under different values of r_m and d_m relative to their wild-type counterparts. Special attention will be paid to the so-called quasi-neutral mutants. We define quasi-neutral mutants as those satisfying

$$r_m = \tau r_w, \quad d_m = \tau d_w. \tag{2}$$

That is, the mutants' rates are proportional to the wild type rates with the factor τ. For $\tau > 1$ the mutants are characterized by a faster turnover, while for $\tau < 1$ they are characterized by a slower turnover.

Here we present some details of calculations focusing on probability of mutant fixation in fragmented and non-fragmented systems, exploring different models of migration and mutant initiation. Supplementary tables 1-3 summarize the notations used here.

1.2. Review of mutant fixation probability in the Moran process

In this work we compare some of our findings to classical results from well-mixed populations, such as the Moran process. These well-mixed results are briefly reviewed here.

Suppose in the Moran process, a mutant has relative fitness $1 + s$ in a population of N individuals. Let us assume that $Ns \gg 1$ and $s \ll 1$ (a large-population, weak selection limit). In the Moran dynamics, the selection coefficient, s, defines the relative probability for a mutant individual to be picked for reproduction to be $(1+s)$, or the relative probability to be picked for death to be $(1 - s)$, depending on whether selection in incorporated into

Quantity	Notation
Division and death rates of wild-type individuals	r_w, d_w
Division and death rates of mutant individuals	r_m, d_m
Carrying capacity of a Verhulst deme	K
Mean population in a deme populated by wild-types and mutants	N_w and N_m
Number of demes	D
Turnover factor for quasi-neutral mutants	τ
Selection coefficient for mutants	s

Supplementary Table 1: System parameters and their notations.

Quantity	Notation
Fitness parameter of a mutant individual (no subscript if constant)	f_j
Fitness parameter of a mutant deme (no subscript if constant)	$\mathcal{F}_J$

Supplementary Table 2: Notations for fitness parameters.

Quantity	Notation
Probability of mutant fixation in a single deme, starting with j mutant and i w.t. individuals	π_{ij}
Prob. of mut. fix. in a single deme, starting with 1 mut. and $N_w - 1$ w.t. individuals	ρ_m
Prob. of w.t. fix. in a single deme, starting with 1 w.t. and $N_m - 1$ mutants	ρ_w
Expected prob. of mut. fix. in a single deme, starting with 1 mut. migrating into a population of w.t. individuals at quasi-equilibrium	$\bar{\rho}_m$
Expected prob. of w.t. fix. in a single deme, starting with 1 w.t. migrating into a population of mutants at quasi-equilibrium	$\bar{\rho}_w$
Expected prob. of mut. fix. in a single w.t. deme, when it is first introduced	ρ_m^{init}
Prob. of a mutant deme fixation starting from a single mutant deme (in the coarse-grained model)	P_{deme}
Prob. of mutant fixation in a fragmented multi-deme system, starting with 1 mutant individual	Π_1^{frag}
Prob. of mutant fixation in an equivalent non-fragmented system, starting with 1 mutant individual	Π_1^{non}

Supplementary Table 3: Notations for fixation probabilities.

birth or death events. The probability of mutant fixation starting from 1 mutants and $N-1$ wild-type individuals is given by [4, 5]

$$\pi_1^{Moran} = \frac{1-(1+s)^{-1}}{1-(1+s)^{-N}} = \frac{1-(1+s)^{-1}}{1-e^{-N\ln(1+s)}} \tag{3}$$

(note that this expression does not apply to quasi-neutral mutants in the context of the Moran process, see [6] for more details). Here we consider the large-population limits of this expression for disadvantageous and advantageous mutants.

- **Disadvantageous mutants,** $s < 0$. We have $\ln(1+s) < 0$ in equation (3). Since $N \gg 1$, one can be neglected compared to $e^{N|\ln(1+s)|}$. Using $1-(1+s)^{-1} \approx s$, we obtain
$$\pi_1^{Moran} \approx s\, e^{-N|\ln(1+s)|}.$$
In other words, the probability of disadvantageous mutant fixation decays exponentially with N.

- **Advantageous mutants,** $s > 0$. Now $\ln(1+s) > 0$ and since $N \gg 1$, $e^{-N\ln(1+s)}$ can be neglected compared to 1 in equation (3). Again using $1-(1+s)^{-1} \approx s$, we obtain
$$\pi_1^{Moran} \approx s,$$
that is, the probability of advantageous mutant fixation in large populations tends to a constant (N-independent) value given by its selection coefficient.

We note that the latter point is related to a result of Haldane [7], also derived by Kimura [8], who writes that "the probability of ultimate survival of an advantageous mutant gene is approximately twice the selection coefficient", $2s$. Kimura's probability of mutant fixation starting with frequency $p = 1/(2N)$ is given by
$$\frac{1-e^{-2s}}{1-e^{-4Ns}},$$
such that for $s < 0$ the probability of fixation decays with N exponentially, while for $s > 0$ it tends to a constant. The factor of 2 difference comes from the difference in the underlying process: the Wright-Fisher formulation used by Haldane and Kimura and the Moran formulation used here. Both expressions for the advantageous mutant fixation (given by $2s$ for the Wright-Fisher and by s for the Moran processes) are presented in equation (12) in [9].

1.2. Probability of mutant fixation in a single deme

1.1. Model formulation and the quasi-stationary probability distribution

Consider a death and birth process where the deaths and the births are decoupled, resulting in demographic fluctuations in the population size. Suppose that parameter K (the carrying capacity) is fixed. Denote the states of the system by a pair of integers (i, j) where i is the number of wild-type individuals and j is the number of mutants. In a version of the Verhulst process with density controlled divisions, we assume that the following processes can occur

during an infinitesimal time-interval, Δt:

$$\begin{aligned}
&\text{w.t. division} && P_{(i,j)\to(i+1,j)} = ir_w\left[1-\frac{i+j}{K}\right]_+\Delta t, && (4)\\
&\text{mut. division} && P_{(i,j)\to(i,j+1)} = jr_m\left[1-\frac{i+j}{K}\right]_+\Delta t, && (5)\\
&\text{w.t. death} && P_{(i,j)\to(i-1,j)} = id_w\Delta t, && (6)\\
&\text{mut. death} && P_{(i,j)\to(i,j-1)} = jd_m\Delta t, && (7)\\
&\text{no change} && P_{(i,j)\to(i,j)} = 1-\left((ir_w+jr_m)\left[1-\frac{i+j}{K}\right]_+(id_w+jd_m)\right)\Delta t, && (8)
\end{aligned}$$

with all the other transition probabilities assumed to be zero. Here $[z]_+$ denotes the positive part,

$$[z]_+ = \begin{cases} z, & z \geq 0, \\ 0, & z < 0. \end{cases}$$

This process has only one absorbing state, $(i,j) = (0,0)$, which is the total population extinction. For our purposes, however, we will assume that extinction takes a very long time. Intuitively, provided that the death rates are not too close to the division rates, K is sufficiently large, and the initial condition is around $N = K(1-d_w/r_w)$, we can ignore the possibility of population extinction on the time-scales of interest (see [3, 10] for the estimate of the extinction time, given the kinetic parameters).

The quasi-stationary probability distribution. To study the system, it is useful to calculate the quasi-stationary probability distribution of the deme size (see also [3]). In a deme with wild-type individuals only, denote the probability of finding the system in state i as P_i. We have

$$d_wP_1 = 0, \tag{9}$$

$$d_wP_22 - P_1\left(r_w\left(1-\frac{1}{K}\right)+d_w\right) = 0, \tag{10}$$

$$r_wP_{i-1}(i-1)\left(1-\frac{i-1}{K}\right) + d_wP_{i+1}(i+1) - P_ii\left(r_w\left(1-\frac{i}{K}\right)+d_w\right) = 0, \quad 1<i<K, \tag{11}$$

$$r_wP_{K-1}(K-1)\left(1-\frac{K-1}{K}\right) - P_KKd_w = 0. \tag{12}$$

It follows that all the probabilities except P_0 are zero. Therefore, in order to obtain the long-term distribution in the absence of extinction, we remove the transition from state 1 to state 0, such that equation (10) for $i = 1$ becomes

$$d_wP_22 - P_1r_w\left(1-\frac{1}{K}\right) = 0, \tag{13}$$

and equation (9) becomes an identity. This gives rise to a new system (13,11,12), where equation (13) replaces equations (9,10) and corresponds to $i = 1$, while equation (11) is for

$1 < i < K$, and equation (12) is for $i = K$. It can be solved together with the normalization $\sum_{i=1}^{K} P_i = 1$ and yields a stationary probability distribution that correctly describes results of numerical simulations, see Supplementary figure 1. Note that the operation of cutting the transition to zero is equivalent to the operation of adding a flux from state 0 to state 1 in this context and leads to the same solution for $P_1, \ldots, P_K$.

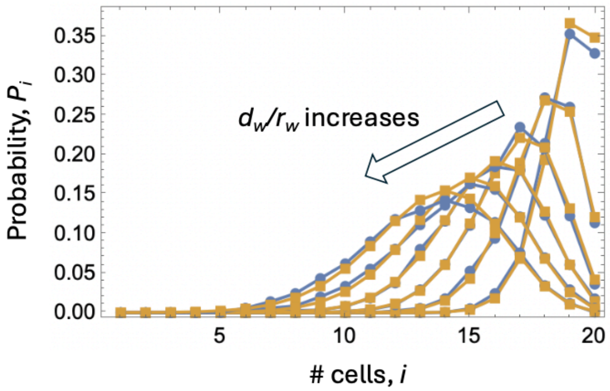

Supplementary Figure 1: Stationary probability distributions, calculated by solving system (13,11,12) (yellow) and obtained from running stochastic simulations (blue). Results for $d_w/r_w = 0.1, 0.2, \ldots, 0.6$ are shown; $K = 20$.

Probability for a deme to be non-full. A useful quantity related to this calculation is the probability of a deme to be non-full (we will use this quantity when studying migration of individuals from deme to deme). Denote by $P_w^{non-full}$ the probability that the population of a deme at a quasi-equilibrium state is less than K. We have

$$P_w^{non-full} = 1 - P_K, \tag{14}$$

where P_K is a component of the normalized solution of system (13,11,12). The probability for a deme to be non-full increases with the death-to-division rate and the carrying capacity, and can be approximated by

$$P_w^{non-full} \approx 1 - \exp\left(-\frac{K^2}{K-1}\frac{d_w}{r_w}\right), \tag{15}$$

as shown in Supplementary figure 2. In particular, two limits are of interest:

- *High-density limit.* If $d_w/r_w \ll 1/K$ (we neglected 1 compared to K in this condition), we have
$$P_w^{non-full} \approx K\frac{d_w}{r_w}. \tag{16}$$
- *Low-density limit.* If $d_w/r_w \gg 1/K$, we have
$$P_w^{non-full} \approx 1. \tag{17}$$

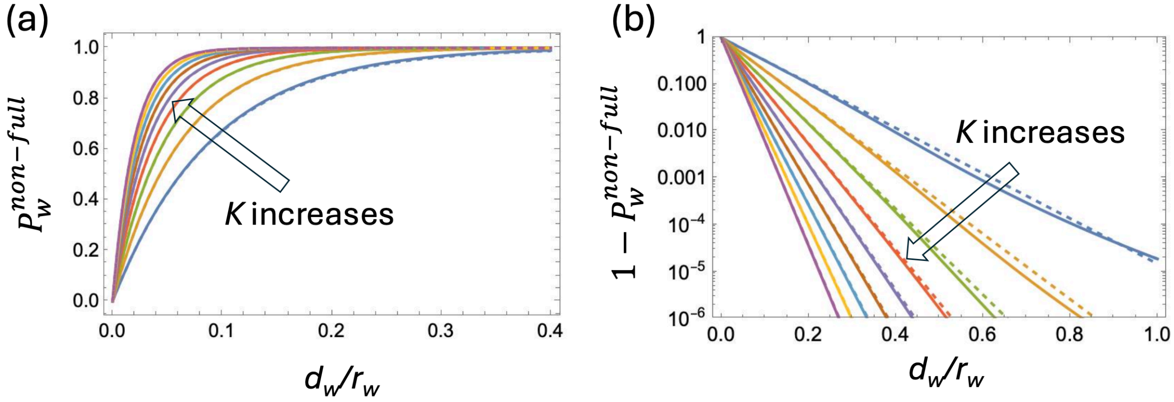

Supplementary Figure 2: The probability for a deme to be non-full. (a) $P_w^{non-full}$ plotted as a function of the death-to-division ratio, d_w/r_w. (b) $1 - P_w^{non-full}$ vs d_w/r_w, plotted on a logarithmic scale. The different colors correspond to different values of the carrying capacity parameter, which was taken to be $10, 15, 20, \ldots, 50$. Solid lines represent formula (14), obtained by explicitly solving system (13,11,12). Dashed lines are approximation (15).

1.2. Calculating mutant fixation probability in a deme

To study the probability of mutant fixation in a deme, let us artificially stop the process once one of the boundary states is reached, that is, we will make all the states $(i, 0)$ with $i \geq 0$ and all the states $(0, j)$ with $j > 0$ absorbing. Then, we will say that "mutants reached fixation" if one of the states $(0, j)$ with $j > 0$ is reached. In the context of the original process, this means that we are interested in the probability of the wild-type individuals going extinct first (with eventually the whole population going extinct).

Denote by π_{ij} the probability of mutant fixation (with the above definition) starting from i wild type and j mutant individuals. This quantity satisfies the following equations:

$$ir_w\left(1-\frac{i+j}{K}\right)\pi_{i+1,j} + jr_m\left(1-\frac{i+j}{K}\right)\pi_{i,j+1} + id_w\pi_{i-1,j} + jd_m\pi_{i,j-1}$$
$$-\left((ir_w+jr_m)\left(1-\frac{i+j}{K}\right)+(id_w+jd_m)\right)\pi_{ij} = 0, \quad i>0, j>0, i+j<K, \tag{18}$$
$$id_w\pi_{i-1,j} + jd_m\pi_{i,j-1} - (id_w+jd_m)\pi_{ij} = 0, \quad i+j \geq K \tag{19}$$

with the boundary conditions given by

$$\pi_{k,0} = 0, \quad \pi_{0,k} = 1, \quad k > 0. \tag{20}$$

System (18-20) can be solved recursively up to an arbitrary value of $i + j = K_0 \geq K$. In particular, of interest is the probability of mutant fixation starting from its quasi-stationary state, where the population is approximately at its deterministic equilibrium,

$$N_w = \lfloor K(1 - d_w/r_w)\rceil, \tag{21}$$

where $\lfloor\cdot\rceil$ denotes rounding. Denote by ρ_m the probability of mutant fixation starting from a single mutant in a population of wild-type individuals. We have by solving system (18-20),

$$\rho_m = \pi_{N_w-1,1}. \tag{22}$$

In this study we will also need to calculate the probability of a wild-type fixation in a population of mutants, which we denote ρ_w. The quasi-stationary state in a mutant deme is given by

$$N_m = \lfloor K(1 - d_m/r_m)\rceil, \tag{23}$$

and we have

$$\rho_w = \pi_{N_m-1,1}, \tag{24}$$

where we use the solution of system (18-20), where the wild-type coefficients were replaced by the mutant coefficients, $r_w \leftrightarrow r_m$ and $d_w \leftrightarrow d_m$.

1.3. Fixation probability of quasi-neutral mutants: comparison with the diffusion approximation

Let us focus on quasi-neutral mutants, equation (2). Note that for such mutants, $N_w = N_m = N$ (equations (21,23)).

Approximate methods can be applied to obtain the probability of mutant fixation, if the population size is large. In [11, 12], the diffusion approximation for quasi-neutral mutants was used to solve a similar system, with the difference that instead of density-controlled divisions, density-controlled death was implemented. The approximate solution for the probability of mutant fixation obtained for this system is given by $\pi_{N-1,1} \approx \rho_m^{diff}$, where

$$\rho_m^{diff} = \frac{1}{N}\frac{2}{\tau+1}. \tag{25}$$

Remarkably, formula (25) gives the same expression as was obtained for the probability of quasi-neutral mutant fixation in the (constant-population) death-birth Moran process [13]. Similarly, the probability of a wild-type fixation in a mutant deme at quasi-equilibrium is approximated by

$$\rho_w^{diff} = \frac{1}{N}\frac{2}{1/\tau+1}.$$

Numerical simulations confirm that these approximations work well for both the process with density controlled death and the process with density controlled divisions, equations (4-8), as long as the population size is large, that is, $\rho_m \to \rho_m^{diff}$ and $\rho_w \to \rho_w^{diff}$, see Supplementary figure 3.

Here we are interested in the deviation of the probability of mutant and wild-type fixation from expressions ρ_m^{diff} and ρ_w^{diff}. Supplementary figure 3(a) shows the ratio of the mutant fixation probability in a population with demographic fluctuations (system (18-20)) and the corresponding value obtained by diffusion approximation, ρ_m/ρ_m^{diff}. We can see that for $\tau = 10 > 1$, which was used in this figure, this ratio is smaller than 1, and tends to one when the carrying capacity (K) increases and the death-to-division ratio decreases. Supplementary figure 3(b) shows a similar plot for the probability of wild-type fixation. The ratio is generally greater than one ($1/\tau = 1/10 < 1$), and again it approaches one when the carrying capacity increases and the death-to-division ratio decreases. Panel (c) shows quantity

$$\mathcal{F} = \tau\rho_m/\rho_w,$$

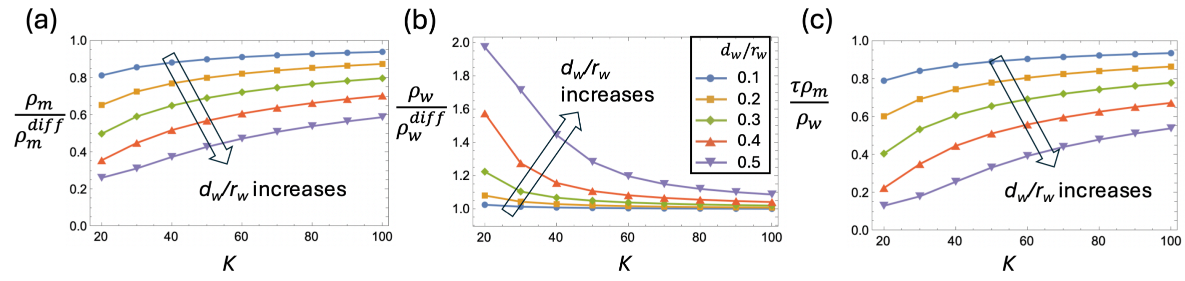

Supplementary Figure 3: Probabilities of fixation in a population with demographic fluctuations (system (18-20)), compared to their values obtained by diffusion approximation, in a system with quasi-neutral mutants. (a) The ratio ρ_m/ρ_m^{diff}, as a function of the carrying capacity, K, plotted for several values of the d_w/r_w ratio. For each point, the population size was taken $N = K(1 - d_w/r_w)$, which is an integer number for all the cases. (b) The same for the ratio ρ_w/ρ_w^{diff}. (c) The same for the quantity $\tau\rho_m/\rho_w$. The ratios d_w/r_w are shown in the inset in panel (b). We used $\tau = 10$ in all panels.

which should be compared with the corresponding quantity under diffusion approximation,

$$\tau\rho_m^{diff}/\rho_w^{diff} = 1.$$

We can see that, for fast mutants ($\tau > 1$) in fluctuating populations, the probability of mutant fixation is smaller than that calculated under diffusion approximation, and the probability of wild-type fixation is larger than that calculated under diffusion approximation. As a result, the ratio ρ_m/ρ_w is smaller than $1/\tau$.

1.3. Probability of mutant fixation in a fragmented system

1.1. The coarse-grained approximation

Next, we consider a system of D demes, where wild-type and mutant individuals can migrate between demes. We assume that a migrating individual is equally likely to land on any deme, in other words, there is no spatial structure in this model. The key assumption is that the migration rate is low compared to the processes within demes, such that typically, after a migration event, the deme populations will become homogeneous (through fixation or extinction of the immigrant type), before the next migration event happens. We will use the coarse grained approximation (see [14]) to calculate the probability of mutant fixation. The key idea is to approximate the probability of mutant fixation, Π_1^{frag}, as

$$\Pi_1^{frag} = \rho_m^{init} P_{deme}, \tag{26}$$

where ρ_m^{init} denotes the expected probability of mutant fixation in a single deme, where it is initially placed, and P_{deme} is the probability of a mutant deme fixation (starting from a single mutant deme, see Supplementary table 3).

To calculate the probability of deme fixation, P_{deme}, consider a collection of D demes, each populated entirely either by mutant or by wild-type individuals. We denote by J the number of mutant demes; the number of wild-type demes is then $D-J$. Let us further denote by W_J^+ the rate, at which the number of mutant demes increases by one, and by W_J^- the

rate, at which the number of mutant demes decreases by one. Denote by h_J the probability of mutant deme fixation starting from J mutant demes. We have, by the standard one-step analysis,

$$0 = W_J^+ h_{J+1} + W_J^- h_{J-1} - h_J(W_J^+ + W_J^-), \quad h_0 = 0, \quad h_D = 1. \tag{27}$$

Denoting

$$\mathcal{F}_J = \frac{W_J^+}{W_J^-},$$

equation (27) can be rewritten as

$$0 = \mathcal{F}_J h_{J+1} + h_{J-1} - h_J(\mathcal{F}_J + 1), \quad h_0 = 0, \quad h_D = 1. \tag{28}$$

The next steps are to construct the expressions for W_J^+, W_J^-, and $\mathcal{F}_J$, solve equation (28), and determine

$$P_{deme} = h_1. \tag{29}$$

1.2. Deme fitness for different migration models

A number of modeling assumptions are possible for the process of migration. The first choice to make is the mechanism, by which migration is carried out. Here we distinguish two major types of migration models:

(1) **Reproduction-independent migration.** We can assume that migration happens as a separate event (distinct from birth and death events), and the migration rate is either the same for both types, or it is proportional to the division rate of individuals.

(2) **Migration by division.** Alternatively, we could assume that migration is incorporated in divisions, when with a given small probability (ϵ) the offspring of a divided individual is transported to a different deme.

As before, we assume that migration rate is low, such that the demes are homogeneous most of the time.

The second modeling choice concerns the migration event destination. Two possibilities are considered here:

(a) **Crowd-controlled migration:** Migration can only occur to a deme whose population size is smaller than K. If the target deme is full (size K) then the migration event is aborted.

(b) **Crowd-independent migration:** Migration happens regardless of whether or not a deme is full.

Below we calculate the rate, at which the number of mutant demes increases by one (W_J^+) and decreases by one (W_J^-), and the resulting expression for the mutant deme fitness. Despite this variety of modeling choices, as we show, the resulting expression for the deme fitness is the same or similar in many cases.

Consider the population of J mutant demes and $D-J$ wild-type demes. The populations of demes fluctuate around their equilibrium values. Wild type and mutant individuals can migrate between demes. A change in the number of mutant and wild-type demes happens through the process of deme conversion. The rate of conversion is given by the rate at which an individual of a given type lands on a deme of a different type, times the probability of fixation of the migrant. We have, in general,

$$
\begin{aligned}
W_J^+ &\propto \text{(net mut. migr. rate)} \times \text{(prob. to land on a w.t. deme)} \times \text{(prob. to fixate there)}, \\
W_J^- &\propto \text{(net w.t. migr. rate)} \times \text{(prob. to land on a mut. deme)} \times \text{(prob. to fixate there)}.
\end{aligned}
$$

We will begin by assuming that the migration process is **reproduction-independent** (choice (1) above) and **crowd-controlled** (choice (a) above). As before, denote by N_w and N_m the equilibrium value of the wild-type and mutant demes, see equations (21,23). We have, for the rates of increase and decrease in the number of mutant demes,

$$
W_J^+ \propto JN_m\mu_m \times \frac{D-J}{D} \times P_w^{non-full} \times \bar{\rho}_m, \tag{30}
$$

$$
W_J^- \propto (D-J)N_w\mu_w \times \frac{J}{D} \times P_m^{non-full} \times \bar{\rho}_w, \tag{31}
$$

where the right hand side of the expression for W_J^+ is proportional to the propensity of mutants to migrate (which is the total number of mutants, JN_m, times the migration rate, μ_m), land on a wild-type deme ($\frac{D-J}{D}$), find that the chosen wild-type deme is non-full ($P_w^{non-full}$), and get fixated there ($\bar{\rho}_m$). The expression for W_J^- is constructed similarly. The probability that a wild-type deme is non-full, $P_w^{non-full}$, is given by equation (14). The probability that a mutant deme is non-full, $P_m^{non-full}$, requires switching the parameters r_w, d_w in system (13,11,12) to parameters r_m, d_m.

From these equations, the fitness parameter for mutant demes is given by

$$
\mathcal{F}_J = \frac{W_J^+}{W_J^-} = \frac{N_m P_w^{non-full}}{N_w P_m^{non-full}} \frac{\mu_m \bar{\rho}_m}{\mu_w \bar{\rho}_w} = \mathcal{F}, \tag{32}
$$

which is independent of the number of mutant demes, J.

Different assumptions on the migration process will modify the expressions in the following way. For **migration by division** (choice (2) above), the rate of migration is ϵd_m for mutants and ϵd_w for wild-type individuals (because at the equilibrium, the rate of successful divisions is equal to the rate of death). These expressions will replace the rates μ_m and μ_w, respectively, in formulas (30) and (31). For **crowd-independent migration** (choice (b) above), the rate of migration does not depend on whether or not the target deme is full. Therefore, expressions $P_m^{non-full}$ and $P_w^{non-full}$ should be replaced by 1 in formulas (30) and (31).

Further, the expressions for the expected probabilities of fixation in a single deme, $\bar{\rho}_m$ and $\bar{\rho}_w$, depend on migration assumptions:

- Crowd independent migration. In this case, $\bar{\rho}_m$ is given by

$$
\bar{\rho}_m = \sum_{i=1}^{K} P_i \pi_{i,1}, \tag{33}
$$

where the values $\pi_{i,1}$ are obtained by solving system (18-20). The expression for $\bar{\rho}_w$ is also given by the right hand side of equation (33), except now the values $\pi_{i,1}$ are solutions of system (18-20), where we swap the mutant and wild-type kinetic rates: $r_w \leftrightarrow r_m$ and $d_w \leftrightarrow d_m$.

- Crowd controlled migration. Since the migration events that target a deme with a population at or above carrying capacity are canceled, we have

$$\bar{\rho}_m = \frac{\sum_{i=1}^{K-1} P_i \pi_{i,1}}{\sum_{i=1}^{K-1} P_i},$$

where again $\pi_{i,1}$ are obtained from system (18-20), and the calculation adjusted for $\bar{\rho}_w$ in the same way as before.

	(1) Reproduction-independent migration	(2) Migration by division
(a) Crowd-controlled migration	$\frac{N_m P_w^{non-full}}{N_w P_m^{non-full}} \frac{\mu_m \bar{\rho}_m}{\mu_w \bar{\rho}_w}$	$\frac{N_m P_w^{non-full}}{N_w P_m^{non-full}} \frac{d_m \bar{\rho}_m}{d_w \bar{\rho}_w}$
(b) Crowd-independent migration	$\frac{N_m}{N_w} \frac{\mu_m \bar{\rho}_m}{\mu_w \bar{\rho}_w}$	$\frac{N_m}{N_w} \frac{d_m \bar{\rho}_m}{d_w \bar{\rho}_w}$

Supplementary Table 4: Expressions for the mutant deme fitness, $\mathcal{F}$, for different assumptions on the migration process.

Supplementary table 4 lists all the combinations of the assumptions. Note that the expressions presented in the table are quite general, in the sense that the microscopic assumptions on the within-deme dynamics can vary. In the model implemented here (that is, a Verhulst process with density-dependent divisions), the expected population sizes of demes are given by equations (21,23), the probability to be non-full is equation (14), obtained by solving system (13,11,12), and the probability of fixation is given by equation (33). Similar expressions can be derived for a more general birth-death process in the demes, where per-individual division and death rates are some functions of the numbers of wild-type and mutant individuals. The present theory requires that the demes' populations fluctuate around a finite quasi-equilibrium size, N_w and N_m, and (for the case of migration by division) that the two species have an intrinsic division rate (what we called r_w, d_w).

1.3. Special cases: high-density mode, low-density mode, and quasi-neutral mutants

High-density mode. As follows from section 1.1, we can use approximations for the quantities $P_w^{non-full}$ and $P_m^{non-full}$. In particular, if the death-to-division ratios satisfy $\frac{d_w}{r_w} \ll \frac{1}{K}$, $\frac{d_m}{r_m} \ll \frac{1}{K}$, the demes are characterized by high density and are at capacity most of the time. Therefore, the equilibrium deme sizes satisfy $N_m \approx N_w \approx K$. Further, using approximation (16) and a similar approximation for $P_m^{non-full}$ in the expressions of deme fitness, we have $P_w^{non-full} \approx K \frac{d_w}{r_w}$, $P_m^{non-full} \approx K \frac{d_m}{r_m}$, and instead of Supplementary table 4, we have simplified expressions for mutant deme fitness, that are summarized in Supplementary table 5.

	(1) Reproduction-independent migration	(2) Migration by division
(a) Crowd-controlled migration	$\frac{d_w r_m}{r_w d_m} \frac{\mu_m \bar{\rho}_m}{\mu_w \bar{\rho}_w}$	$\frac{d_w r_m}{r_w d_m} \frac{d_m \bar{\rho}_m}{d_w \bar{\rho}_w}$
(b) Crowd-independent migration	$\frac{\mu_m \bar{\rho}_m}{\mu_w \bar{\rho}_w}$	$\frac{d_m \bar{\rho}_m}{d_w \bar{\rho}_w}$

Supplementary Table 5: High-density mode, where $\frac{d_w}{r_w} \ll \frac{1}{K}$, $\frac{d_m}{r_m} \ll \frac{1}{K}$. Expressions for the mutant deme fitness, $\mathcal{F}$, are given for different assumptions on the migration process.

Low-density mode. In the opposite limit, where the death-to-division ratios satisfy $\frac{d_w}{r_w} \gg \frac{1}{K}$, $\frac{d_m}{r_m} \gg \frac{1}{K}$, the demes are characterized by low density and rarely reach the carrying capacity. As a consequence, using approximation (17) and a similar approximation for $P_m^{non-full}$ in the expressions of deme fitness, we obtain:

$$\text{(low-density:)} \quad \mathcal{F} = \begin{cases} \frac{N_m}{N_w} \frac{\mu_m \bar{\rho}_m}{\mu_w \bar{\rho}_w}, & \text{reproduction-independent migration,} \\ \frac{N_m}{N_w} \frac{d_m \bar{\rho}_m}{d_w \bar{\rho}_w}, & \text{migration by division.} \end{cases}$$

In other words, since the demes are almost never full, there is no difference between crowd-controlled and crowd-independent migration models in this case.

Mutant deme fitness for quasi-neutral mutants. The expressions for mutant deme fitness (regardless of the population density) simplify significantly in the case of quasi-neutral mutants. Since the equilibrium deme sizes (equations (21,23)) and the solution of system (13,11,12) only depend on the ratio, d_w/r_w, for quasi-neutral mutants we have

$$\text{(quasi-neutral:)} \quad N_m = N_w, \quad P_m^{non-full} = P_w^{non-full}.$$

Therefore, for the deme fitness, we obtain,

$$\text{(quasi-neutral:)} \quad \mathcal{F} = \begin{cases} \frac{\mu_m \bar{\rho}_m}{\mu_w \bar{\rho}_w}, & \text{reproduction-independent migration,} \\ \tau \frac{\bar{\rho}_m}{\bar{\rho}_w}, & \text{migration by division.} \end{cases}$$

If the migration rate in the reproduction-independent migration is taken to be proportional to the division rate, $\mu_m/\mu_w = r_m/r_w$, then the two cases coincide, yielding $\mathcal{F} = \tau \frac{\bar{\rho}_m}{\bar{\rho}_w}$. A similar quantity was studied in Section 1.3, see e.g. Supplementary figure 3(c).

1.4. Probability of deme fixation and different initiation models

To calculate the probability of mutant fixation in a fragmented population by using coarse-grain approximation, equation (26), we need to calculate the probability of mutant deme fixation, P_{deme}. Since the fitness of a deme, $\mathcal{F}$, is a quantity independent of J, solution of equation (28) is given by $h_J = \frac{1-1/\mathcal{F}^J}{1-1/\mathcal{F}^D}$, and therefore from (29) we have

$$P_{deme} = \frac{1 - 1/\mathcal{F}}{1 - 1/\mathcal{F}^D}. \tag{34}$$

Apart from using different migration models, there is also a choice of ways in which the system can be initialized. We consider several cases here, all of which are described by equation (26), where we used equation (34):

$$\Pi_1^{frag} = \rho_m^{init} \frac{1 - 1/\mathcal{F}}{1 - 1/\mathcal{F}^D}.$$

Here ρ_m^{init} stands for the probability of mutant fixation in the deme where it was initially placed, and the mutant deme fitness is calculated in sections 1.2 and 1.3. The deme fitness calculations involve quantities $\bar{\rho}_m$ and $\bar{\rho}_w$, which are probabilities of mutant and wild-type fixation upon migration, with $\bar{\rho}_m$ that may or may not be different from ρ_m^{init}.

The initial probability of fixation, ρ_m^{init}, depends on the way the first mutant is placed. In the literature, several models are used, see e.g. [15]:

(i) Uniform (by deme) mutant initialization, where one individual in a uniformly randomly chosen wild-type deme is turned into a mutant. In this case, we have

$$\rho_m^{init} = \sum_{i=1}^{K} P_i \pi_{i-1,1}.$$

(ii) Uniform (by individual) mutant initialization, where a uniformly randomly chosen wild-type individual is turned into a mutant. In this case, we have

$$\rho_m^{init} = \frac{\sum_{i=1}^{K} P_i i \pi_{i-1,1}}{\sum_{i=1}^{K} P_i i}.$$

(iii) Temperature mutant initialization, where the initial mutant is assumed to be produced by a dividing individual. We have

$$\rho_m^{init} = \frac{\sum_{i=1}^{K-1} P_i i(1 - i/K)\pi_{i,1}}{\sum_{i=1}^{K-1} P_i i(1 - i/K)}.$$

(iv) A mutant is introduced by an external migration event. In this case, we set $\rho_m^{init} = \bar{\rho}_m$, where the latter quantity is calculated as described above, in accordance with the migration model used.

1.5. Probability of quasi-neutral mutant fixation in fragmented populations and their effective fitness

In Fig. 1 of the main paper, as well as in the examples below, we assume that migration by division is crowd-controlled (model (2a) of section 1.2), and that the initialization happens according to scenario (iv) of section 1.4.

Supplementary figure 4 is similar to Fig. 1(A-C) of the main paper, as it shows the probability of quasi-neutral mutant fixation in a fragmented system as a function of (a) the

total population size, by increasing the number of demes, (b) the turnover factor, τ, and (c) the death-to-division ratio, d_w/r_w. The difference compared to the main text figure is that here we explore mutants that experience a slowing in their turnover, that is, $\tau < 1$. The values Π_1^{frag} are calculated as explained above (see the solid lines). They are compared with the corresponding functions for the non-fragmented populations, $\Pi_1^{non} = \frac{1}{ND}\frac{1}{\tau+1}$ (the dashed lines). We can see that the fixation probability in a fragmented system is always larger than that for the non-fragmented population.

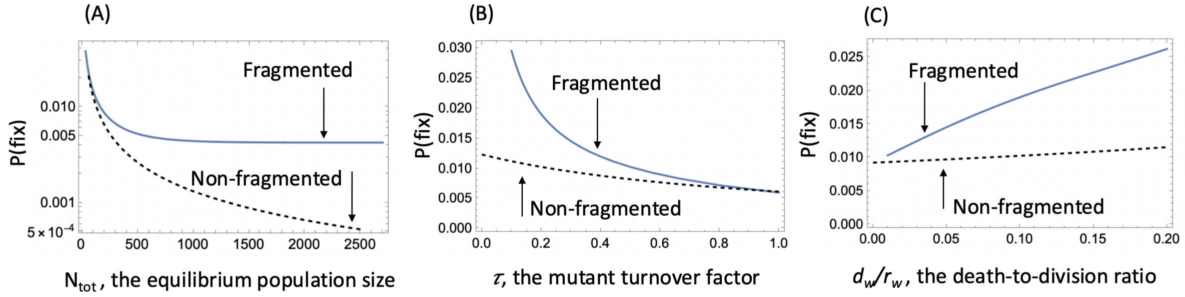

Supplementary Figure 4: Probability of quasi-neutral mutant fixation in the deme-structured model, obtained by the coarse-grained theory, with $\tau < 1$. The solid line is the quantity Π_1^{frag}, and the dashed line is Π_1^{non}. (A) The mutant fixation probability increases and reaches a saturation as a function of the total quasi-equilibrium population size, $N_{tot} = ND$, a characteristic of an advantageous mutant. (B) The mutant fixation probability declines with the amount by which the birth and death rates are proportionally increased, τ. (C) The increase of the mutant fixation probability in the deme-structured compared to the non-fragmented population becomes more substantial with higher death-to-birth ratios. Base parameters were chosen as follows: For (A) $\tau = 0.5$; D varies from 2 to 150. For (B) $D = 9$. For (C) $\tau = 0.2, D = 9$. The rest of the parameters are $d_w/r_w = 0.1; \epsilon = 10^{-4}; K = 20; s = 0$.

We also observe a qualitative difference between the behavior of Π_1^{frag} and Π_1^{non}, as we increase the number of demes, D (and thus the total population size, $N_{tot} = DN$). The probability of quasi-neutral mutant fixation in a non-fragmented population, Π_1^{non}, is always inversely proportional to the total population size. On the other hand, Π_1^{frag} decreases exponentially with N_{tot} if $\tau > 1$ (see Fig. 1(A) of the main paper), and it increases and reaches a constant value for $\tau < 1$ (see Supplementary figure 4(a)). This behavior of the quantity Π_1^{frag} suggests that the quasi-neutral mutant in a fragmented populations acquires features of a truly disadvantageous mutant with $\tau > 1$ and a truly advantageous mutant with $\tau < 1$. This can be shown by rewriting the expression for Π_1^{frag} as

$$\Pi_1^{frag} = \rho_m^{init}\frac{1-1/\mathcal{F}}{1-1/\mathcal{F}^D} = \alpha\frac{1-1/f_e}{1-1/f_e^{N_{tot}}}, \tag{35}$$

where $N_{tot} = ND$, the "effective" fitness parameters of quasi-neutral individuals, f_e, is given by

$$f_e = \mathcal{F}^{1/N}, \tag{36}$$

and the proportionality constant, α, is independent on the number of demes and is defined as

$$\alpha = \rho_m^{init}\left(\frac{1-1/f_e}{1-1/f_e^N}\right)^{-1}.$$

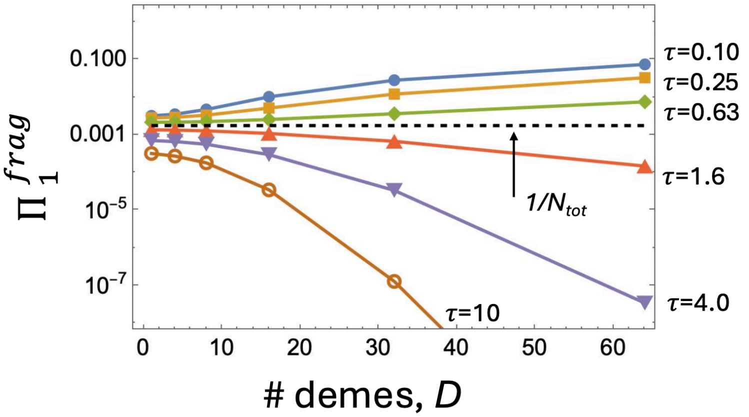

Supplementary Figure 5: The role of fragmentation in quasi-neutral mutant fixation probability. The quantity Π_1^{frag} is plotted as a function of the number of demes, D, for a fixed total carrying capacity, $K_{tot} = 640$. Each curve corresponds to a different turnover parameter τ that ranges from $\tau = 10$ to $\tau = 0.1$, as marked. The probability of neutral mutant fixation in the whole population is shown by the horizontal line. The death-to-division ratio is $d_w/r_w = 0.1$.

The latter expression is the ratio between the probability of the initial mutant to fixate in a single deme, and the probability of a mutant with fitness f_e to fixate in a deme of the same size, if it is subject to the usual Moran dynamics.

Let us consider quasi-neutral mutants in relatively small demes, where deme fitness is not equal to one. If $\tau > 1$, then both $\mathcal{F} < 1$ and $f_e < 1$, then we can see from equation (35) that the fixation probability decays exponentially with the population size, N_{tot}. If on the other hand, $\tau < 1$, then $\mathcal{F} > 1$ and $f_e > 1$, then from equation (35), the fixation probability increases and saturates at the level $\alpha \, \frac{f_e - 1}{f_e}$ as N_{tot} increases. Finally, if $\mathcal{F} = 1$ (which happens if the deme size is large), we have $f_e = 1$, $\rho_m^{init} = \frac{1}{N}\frac{2}{\tau+1}$, $\alpha = \frac{2}{\tau+1}$, and $\Pi_1^{frag} = \frac{2}{\tau+1}\frac{1}{N_{tot}}$.

To summarize, the behavior of quasi-neutral mutants in fragmented populations with small demes of a fixed size resembles that of non-neutral mutants whose effective fitness is given by (36). The probability of mutant fixation depends on the total population size as in equation (35), with a constant factor α independent on the deme number.

1.6. The role of fragmentation in quasi-neutral and non-neutral mutant fixation

Next, we examine the influence of fragmentation on the probability of quasi-neutral mutant fixation, under a constant total populatuon size. In Supplementary figure 5 we split the total system with carrying capacity, K_{tot} into 4,8,16,32, and 64 equal parts; the number of parts is denoted by D as before. Six different values of the turnover parameter, τ, were used. Three of them are greater than one; for those parameters, fragmentation results in a decrease of fixation probability. For $\tau = 10$, when the total population is split into 64 demes, the reduction in fixation probability is enormous (about 10^{-10}). But even for modest values of τ such as $\tau = 1.6$, the reduction in fixation probability reaches about 10-fold for $D = 64$.

Supplementary figure 6 explores the role of fragmentation in fixation dynamics of non-neutral mutants. Two scenarios are presented. In panel (a), the mutants have a division-to-death ratio greater than that of the wild-type (with the selection coefficient $s = 0.01$, which makes them advantageous in a non-fragmented population), but their turnover rate is faster than that for the wild-type. As before, the total carrying capacity is kept constant, and the population is subdivided into 4,8,16,32, and 64 parts. As the degree of fragmentation increases, the probability of mutant fixation decreases and becomes less than that for neutral mutants. In other words, in a subdivided population, a faster turnover rate may turn an advantageous mutant into a disadvantageous mutant.

In panel (b) we consider the opposite scenario, where the mutants have a division-to-death ratio smaller than that of the wild-type (with the selection coefficient $s = -0.01$, which makes them disadvantageous in a non-fragmented population), but their turnover rate is slower than that for the wild-type. As the degree of fragmentation increases, the probability of mutant fixation increases and becomes larger than that for neutral mutants. Therefore, in a subdivided population, a slower turnover rate may turn a disadvantageous mutant into an advantageous mutant.

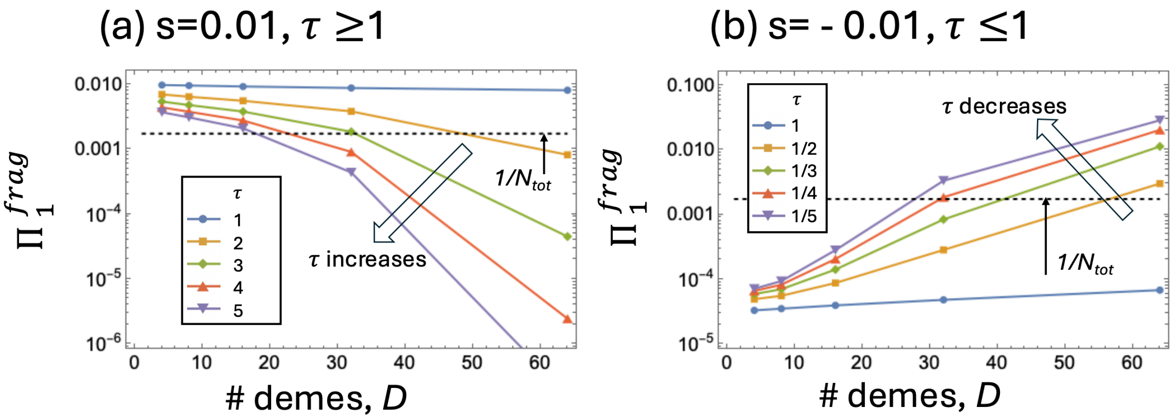

Supplementary Figure 6: The role of fragmentation in non-neutral mutant fixation probability. (a) Advantageous fast mutants: $s > 0, \tau > 1$. (b) Disadvantageous slow mutants: $s < 0, \tau < 1$. The quantity Π_1^{frag} is plotted as a function of the number of demes, D, for a fixed total carrying capacity, $K_{tot} = 640$. The probability of neutral mutant fixation in the whole population is shown by the horizontal line. Different curves correspond to different turnover parameters, as indicated in the legend. The rest of the parameters are $r_w = 0.5, r_m = (1 + s)\tau r_w, d_w = 0.05, d_m = \tau d_w$.

1.4. Comparison with the framework of [16]

Here we recast our approach using the framework of [16]. In the latter paper, Malthusian fitness and the turnover of type i are defined, respectively, as

$$W_i = (\text{birth rate})_i - (\text{death rate})_i, \quad T_i = (\text{birth rate})_i + (\text{death rate})_i,$$

where the two terms are density-dependent per capita division and death rates, and here we employed capital letters W and T, because symbols w and τ are used in our paper for other

quantities. In [16] it was found that the expression for drift in the stochastic dynamics for the frequency of type i contains two terms:

(i) a positive contribution proportional to $(W_i - \bar{W})$, which is the influence of "fitness" in the usual sense of this word, and

(ii) a negative contribution proportional to $(T_i - \bar{T})$, which is the influence of acceleration/deceleration of the turnover

(here $\bar{W}$ and $\bar{T}$ denote the averaged fitness and turnover of all the types, respectively). In particular, in the presence of just two types, the equation for the frequency of the 2nd type (p) contains terms multiplying (i) $(W_2 - W_1)p(1-p)$ and (ii) $\frac{1}{N}(T_2 - T_1)p(1-p)$.

For our model the quantities W and T are given by

$$W_i = r_i \left(1 - \frac{\sum_k x_k}{K}\right) - d_i, \quad T_i = r_i \left(1 - \frac{\sum_k x_k}{K}\right) + d_i,$$

where i takes values "w" and "m" for the wild types and mutants. Let us evaluate the quantities W_i and T_i at the equilibrium corresponding to the wild type: $x_w = K\left(1 - \frac{d_w}{r_w}\right)$, $x_m = 0$. We obtain

$$W_w = 0, \quad W_m = r_m \frac{d_w}{r_w} - d_m = \frac{d_w d_m}{r_w}\left(\frac{r_m}{d_m} - \frac{r_w}{d_w}\right), \quad W_m - W_w = \tau d_w s, \tag{37}$$

$$T_w = 2d_w, \quad T_m = r_m \frac{d_w}{r_w} + d_m, \quad T_m - T_w = 2d_w(\tau - 1) \tag{38}$$

(we assumed $s \ll 1$ in equation (38)). In this study we use two parameters to define the relationship between mutant and wild-type kinetic rates: the selection coefficient, s, and the turnover factor, τ: $r_m = (1+s)\tau r_w$ and $d_m = \tau d_w$. From equations (37) and (38) we can see that the sign of the selection coefficient controls the sign of contribution (i), and whether the turnover factor τ is acceleration ($\tau > 1$) or decelerating ($\tau < 1$) controls the sign of contribution (ii). Further,

- if $s = 0$ (no change in the maximum lifetime reproductive potential: $r_w/d_w = r_m/d_m$), we have (i) $W_m - W_w = 0$ and (ii) $T_m - T_w = 2d_w\tau$, and parameter τ only appears in the turnover term.

- If $\tau = 1$ (no proportional rescaling of kinetic parameters), we have (i) $W_m - W_w = d_w s$ and (ii) $T_m - T_w = 0$, and the selection parameter, s, only enters the Malthusian fitness term.

Supplementary Note 2. Dynamics in the presence of *de novo* mutations

2.1. Selection-mutation balance in fragmented populations

Here we adapt the coarse-grained approach to study the process of *de-novo* mutations in the system. Again we will assume that migration by division is crowd-controlled (model (2a) of section 1.2). The calculations can be easily modified to incorporate the other assumptions discussed in the previous sections.

As before, denote the number of mutant demes by J. In the presence of *de-novo* mutations, the probabilities to increase and decrease the number of mutant demes are given by

$$W_J^+ = JN_m\epsilon d_m \frac{D-J}{D} P_w^{non-full}\bar{\rho}_m + (D-J)N_w d_w u\bar{\rho}_m, \quad (39)$$

$$W_J^- = (D-J)N_w\epsilon d_w \frac{J}{D} P_m^{non-full}\bar{\rho}_w, \quad (40)$$

where the second term in the expression for W_J^+ represents divisions of wild-type cells that result in a mutation (probability u per division). Solving the equation

$$W_J^+ = W_J^-,$$

we can find the value, J, that corresponds to selection-mutation balance:

$$J_{sel-mut} = \frac{Du}{\epsilon P_m^{non-full}(1-\mathcal{F})}, \quad \mathcal{F} = \frac{N_m P_w^{non-full} d_m\bar{\rho}_m}{N_w P_m^{non-full} d_w\bar{\rho}_w} \quad (41)$$

(note that the expression for mutant deme fitness, $\mathcal{F}$, is the same as appears in Supplementary table 4 for this case).

Supplementary figure 7 illustrates this with an example. We ran stochastic simulations in a system of 100 demes with $K = 20$ each, in the presence of rare migrations and mutations. The mutants are assumed to have a negative selection coefficient ($s = -0.01$). Panel (a) of Supplementary figure 7 shows a typical trajectory; the number of mutants in the system undergoes large fluctuations. Finding the temporal mean number of mutants of 516 independent simulations of this type, we plotted a histogram of mean mutant populations (panel (b)). The red vertical line shows the theoretical prediction, $J_{sel-mut}$, obtained by equation (41). We can see that the theory is consistent with the simulations, which is further illustrated in panel (c), where the mean temporal course of 516 simulations is plotted together with the predicted mean number of mutants.

2.2. The reversal of the selection forces as a result of a changing turnover factor

A change in the turnover factor, τ, can lead to qualitative changes in the behavior of a spatial or fragmented system, such that a disadvantageous mutant ($s < 0$) starts behaving as if it

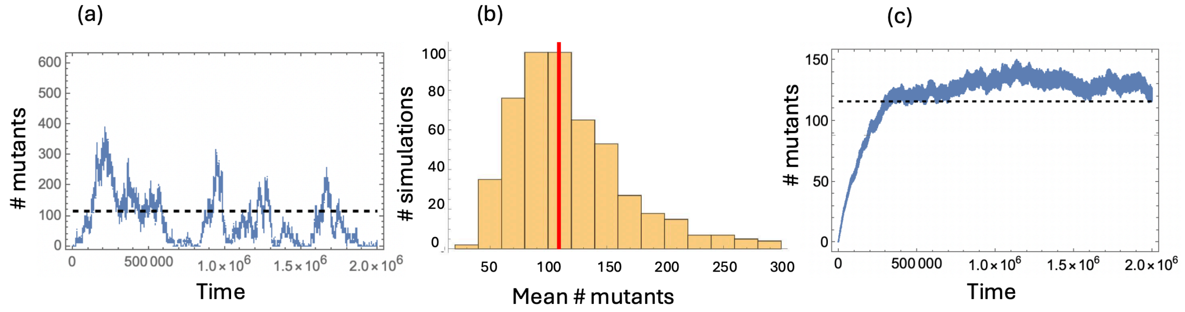

Supplementary Figure 7: Selection-mutation balance. (a) A typical trajectory obtained by Gillespie simulations of a deme-structured population with mutations. The number of mutants is shown as a function of time. (b) A histogram of temporal averages of mutant numbers for 516 independent simulations. (c) Ensemble-averaged time-series for the number of mutants calculated from the 516 simulations, together with the standard error. The red vertical line and the dashed horizontal lines show the theoretical prediction, $J_{sel-mut}$, obtained from eqiation (41). The parameters are $D = 100, K = 20, s = -0.01, r_w = 5, d_w = 0.5, \epsilon = 10^{-4}, u = 10^{-6}, \tau = 1$.

is selected for and takes over the population. This happens if the turnover factor is small enough. To see this, we observe that the expression $(1 - \mathcal{F})$ appears in the denominator of equation (41). An increase of $\mathcal{F}$ (which results from a decrease in τ) increases the level at which mutants are maintained in the system, until $\mathcal{F}$ grows above 1, at which point equation (41) breaks down and the mutant is no longer disadvantageous. This is illustrated in Supplementary figure 8(a), where the singularity of the quantity $J_{sel-mut}$ is observed around $\tau \approx 0.2$.

Similarly, an advantageous mutant that takes over the population in a nearly-deterministic fashion with $\tau = 1$, may become disadvantageous if it is accelerated sufficiently (see panel (b) of Supplementary figure 8).

The value of τ that reverses selection forces is given implicitly by the equation

$$\frac{\bar{\rho}_m}{\bar{\rho}_w} = \frac{N_w P_m^{non-full} d_w}{N_m P_w^{non-full} d_m}, \tag{42}$$

where the right hand side does not depend on τ.

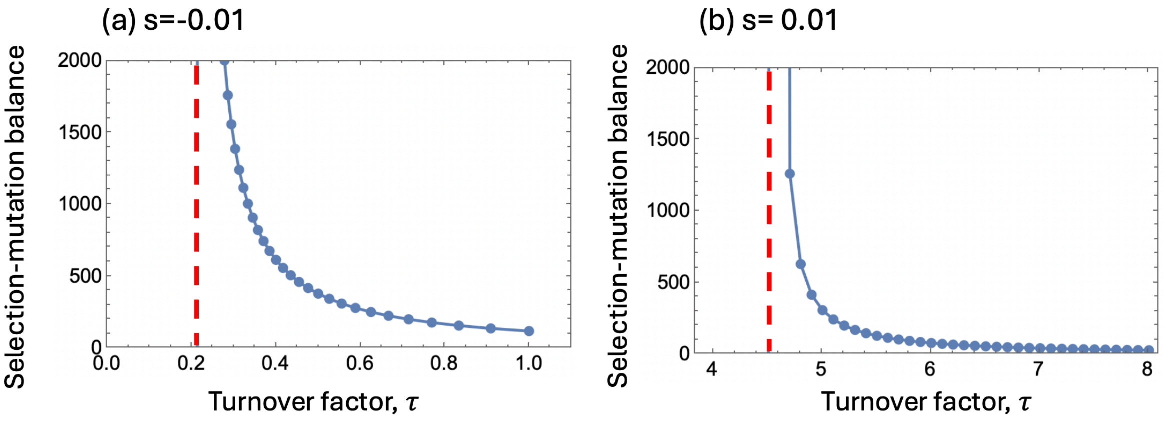

Supplementary Figure 8: The effect of the turnover factor on mutant behavior in the presence of *de novo* mutations. The theoretically calculated selection-mutation balance, $J_{sel-mut}K(1-d_m/r_m)$ (equation (41)) is plotted as a function of τ. (a) Disadvantageous mutants, $s=-0.01$. (b) Advantageous mutants, $s=0.01$. The red vertical line indicates the value of τ that makes the denominator in $J_{sel-mut}$ zero (equation (42)). Other parameters are $K=20, d_w/r_w=0.1, \epsilon=10^{-4}, u=10^{-6}$.

Supplementary Note 3. Agent-based modeling

3.1. Mutant fixation probability

Supplementary figure 9 illustrates the results of ABM simulations of a spatial system. It presents the dependence of the mutant fixation probability in a spatially explicit ABM on various system parameters. This figure should be compared with figure 1(a-c) of the main text, where we show similar results for the fragmented deme model. The yellow line in Supplementary figure 9(c) should be compared with figure 2(a) of the main text.

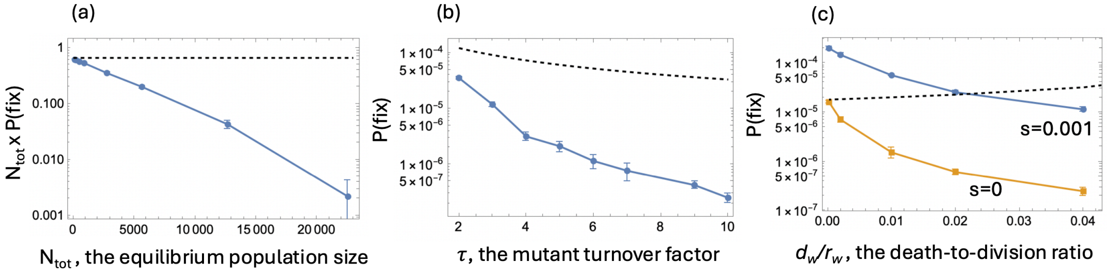

Supplementary Figure 9: Spatial simulation results for the ABM model. In each plot, the mean and standard deviation is presented for each point. The dashed black line is the result for equivalent parameter values in a non-spatial system (with $s=0$). (a) The dependence of $N_{tot}P(\text{fix})$ on the system size, N_{tot}, with $\tau=2$. (b) The dependence of $P(\text{fix})$ on the turnover factor, τ. The equilibrium population size is $N_{tot}=5643$. (c) The dependence of $P(\text{fix})$ on the death-to-division ratio, d_w/r_w, for mutants with $\tau=10$ and $s=0.001$ (blue) and $s=0$ (yellow). In all panels, $d_w/r_w=0.04$ and $s=0$ unless otherwise noted. The total number of independent runs is at least 10^6 in panel (c) with $s=0.001$ and at least 10^7 in the rest of the cases.

3.2. Dynamics in the presence of *de novo* mutations

Supplementary figure 10 shows the reversal of the selection force for mutants in spatially explicit ABM simulations. Panels (a,b) describe mutants with a negative selection coefficient ($s = -0.001$), which fluctuate around a selection-mutation balance if $\tau = 1$ (panel (a)), but take over the population if their division and death rates are slowed down compared to the wild-type by a factor $\tau = 0.1$ (panel (b)). In this case, they act as if they are selected for, although the selection coefficient is negative and the life-time reproductive output of the mutants is smaller than that of the wild types.

Panels (c,d) show the opposite effect. There, the mutants are characterized by a positive selection coefficient ($s = 0.001$), and proceed to take over the population in a typical simulation corresponding to $\tau = 1$ (panel (c)). If however we increase the turnover factor to $\tau = 3$, we observe a qualitative change in the behavior, where mutants are maintained at a selection-mutation balance (panel (d)), despite them being characterized by a higher reproductive outcome compared to the wild-type individuals. Supplementary figure 10 should be compared to figure 3 of the main text, which shows similar effects for a deme-structured population.

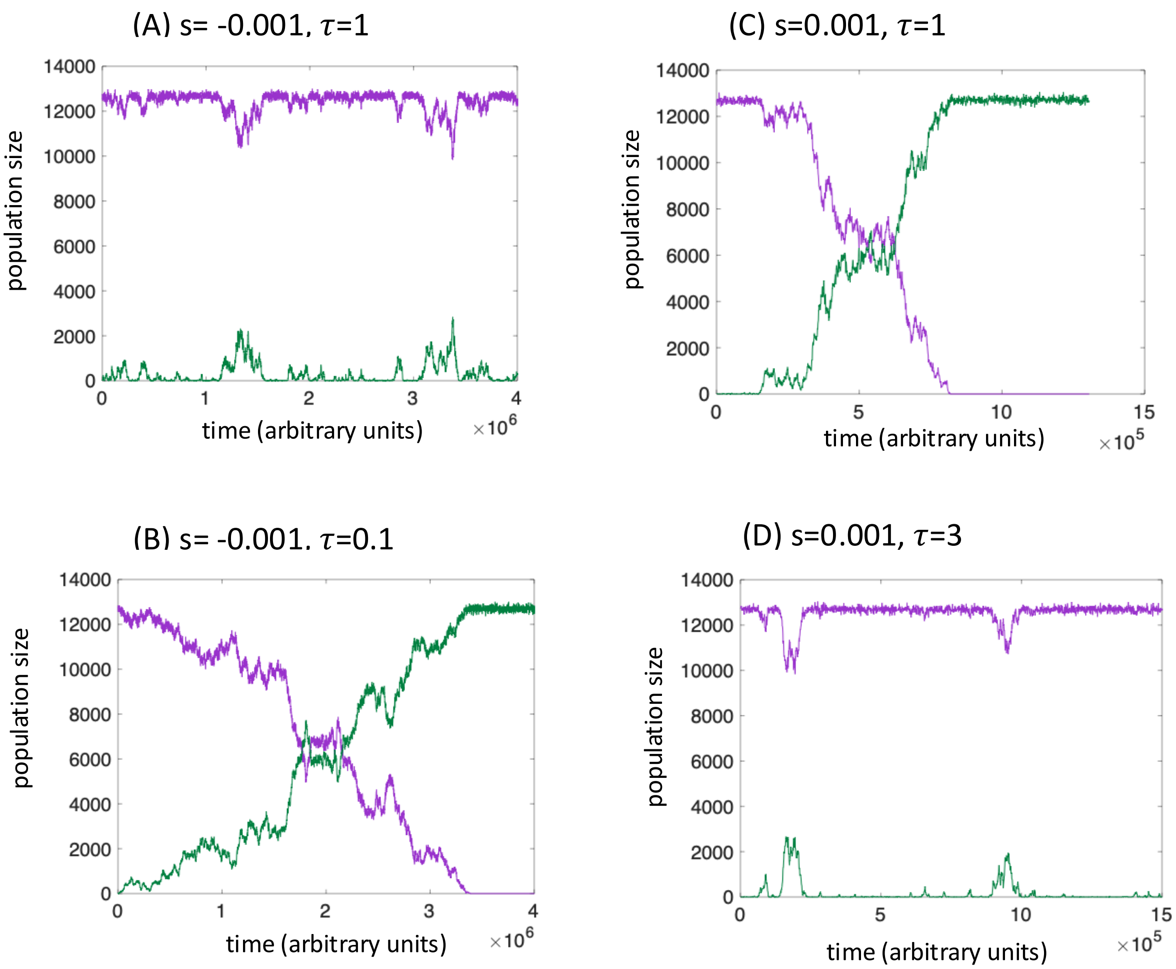

Supplementary Figure 10: The reversal of selection forces in spatial ABM simulations. Time-series of the wild-type (purple) and mutant (green) numbers are shown for typical simulation realizations. (a) $s = -0.001, \tau = 1$ (selection-mutation balance); (b) $s = -0.001, \tau = 0.1$ (mutant takes over); (c) $s = 0.001, \tau = 1$ (mutant takes over); (d) $s = 0.001, \tau = 3$ (selection-mutation balance). Other parameters are $R_w = 0.05, D_w = 0.02, u = 10^{-5}$, and the grid size is 150×150.

Supplementary References

[1] Pierre-François Verhulst. Notice sur la loi que la population suit dans son accroissement. *Correspondence mathematique et physique*, 10:113–129, 1838.

[2] Raymond Pearl and Lowell J Reed. On the rate of growth of the population of the United States since 1790 and its mathematical representation. *Proceedings of the national academy of sciences*, 6(6):275–288, 1920.

[3] Ingemar Nåsell. Extinction and quasi-stationarity in the Verhulst logistic model. *Journal of Theoretical Biology*, 211(1):11–27, 2001.

[4] Patrick Alfred Pierce Moran. Random processes in genetics. In *Mathematical proceedings*

of the Cambridge Philosophical Society, volume 54, pages 60–71. Cambridge University Press, 1958.

[5] Martin A Nowak. *Evolutionary dynamics: exploring the equations of life*. Harvard university press, 2006.

[6] Kamran Kaveh, Natalia L Komarova, and Mohammad Kohandel. The duality of spatial death–birth and birth–death processes and limitations of the isothermal theorem. *Royal Society open science*, 2(4):140465, 2015.

[7] John Burdon Sanderson Haldane. A mathematical theory of natural and artificial selection, part V: selection and mutation. In *Mathematical proceedings of the Cambridge Philosophical Ssociety*, volume 23, pages 838–844. Cambridge University Press, 1927.

[8] Motoo Kimura. On the probability of fixation of mutant genes in a population. *Genetics*, 47(6):713, 1962.

[9] Arthur Alexandre, Alia Abbara, Cecilia Fruet, Claude Loverdo, and Anne-Florence Bitbol. Bridging Wright–Fisher and Moran models. *Journal of Theoretical Biology*, 599:112030, 2025.

[10] Charles R Doering, Khachik V Sargsyan, and Leonard M Sander. Extinction times for birth-death processes: Exact results, continuum asymptotics, and the failure of the Fokker–Planck approximation. *Multiscale Modeling & Simulation*, 3(2):283–299, 2005.

[11] Todd L Parsons and Christopher Quince. Fixation in haploid populations exhibiting density dependence II: The quasi-neutral case. *Theoretical population biology*, 72(4):468–479, 2007.

[12] Todd L Parsons, Christopher Quince, and Joshua B Plotkin. Some consequences of demographic stochasticity in population genetics. *Genetics*, 185(4):1345–1354, 2010.

[13] Dominik Wodarz, Ajay Goel, and Natalia L Komarova. Effect of cell cycle duration on somatic evolutionary dynamics. *Evolutionary Applications*, 10(10):1121–1129, 2017.

[14] Dominik Wodarz and Natalia L Komarova. Mutant fixation in the presence of a natural enemy. *Nature Communications*, 14(1):6642, 2023.

[15] Ben Adlam, Krishnendu Chatterjee, and Martin A Nowak. Amplifiers of selection. *Proceedings of the Royal Society A: Mathematical, Physical and Engineering Sciences*, 471(2181):20150114, 2015.

[16] Ananda Shikhara Bhat and Vishwesha Guttal. Eco-evolutionary dynamics for finite populations and the noise-induced reversal of selection. *The American Naturalist*, 205(1):1–19, 2025.
