# Supplementary material for: Population structure reverses selection of variants with proportionally scaled birth and death rates
Source: Nat Commun. 2025 Dec 27;17:244. doi: 10.1038/s41467-025-66951-x (PMC12783849; doi:10.1038/s41467-025-66951-x)
Supplement: Supplementary file 2 — Description of Additional Supplementary File [file 41467_2025_66951_MOESM2_ESM.pdf]

Description of Additional Supplementary File

Supplementary Code 1: (a) deme-fixprob.f90. This is the Fortan code that numerically determines the fixation probabilities of mutants in the deme-structured model. It is meant to be run in parallel across a computer cluster, and takes the file name for each run as a command line argument. The outputs of the different runs have to be then assembled to calculate the overall fixation probability. **(b) abm-fixprob.f90.** This is the Fortran code that numerically determines the fixation probabilities of mutants in the agent-based model. It is meant to be run in parallel across a computer cluster, and takes the file name for each run as a command line argument. The outputs of the different runs have to be then assembled to calculate the overall fixation probability.
